# Evaluation of Long-Term Outcomes of Crohn’s Disease Complicated by Intra-Abdominal Abscess: A Retrospective International Cohort Study

**DOI:** 10.3390/jcm15072724

**Published:** 2026-04-03

**Authors:** Péter Bacsur, Sylwia Nemeczek, Rafał Filip, Fotios Fousekis, Konstantinos Mpakogiannis, Anna Kagramanova, Konstantinos Argyriou, Ploutarchos Pastras, Christos Triantos, Pál Miheller, María José Casanova, María Chaparro, Andreas Blesl, Sophie Vieujean, Ákos Iliás, Lóránt Gönczi, Murat Toruner, Marko Brinar, Yvette Gatt, Magdalena Gawon-Kiszka, János Tajti, György Lázár, Tamás Resál, Bernadett Farkas, Noémi Gálfalvi, Máté Pápista, Peter L. Lakatos, Klaudia Farkas, Tamás Molnár

**Affiliations:** 1 Department of Medicine, Albert Szent-Györgyi Medical School, University of Szeged, Kálvária Ave. 57, H-6725 Szeged, Hungary; 2 Translational Colorectal Research Group, Hungarian Centre of Excellence for Molecular Medicine (HCEMM), University of Szeged (USZ), H-6728 Szeged, Hungary; 3 Inflammatory Bowel Diseases Unit, Department of Gastroenterology, Clinical Hospital No. 2, 35-301 Rzeszow, Poland; sylwianem@gmail.com (S.N.);; 4 Institute of Medicine, Medical College, Rzeszow University, 35-959 Rzeszow, Poland; 5 Division of Gastroenterology, Department of Internal Medicine, Faculty of Medicine, School of Health Sciences, University of Ioannina, 45110 Ioannina, Greece; fotisfous@gmail.com (F.F.);; 6 Moscow Clinical Scientific Center Named After A.S. Loginov, 111123 Moscow, Russia; 7 Research Institute of Health Organization and Medical Management, 115088 Moscow, Russia; 8 Inflammatory Bowel Diseases Unit, Department of Gastroenterology, University Hospital of Larissa, 41110 Larissa, Greece; kosnar2@yahoo.gr; 9 Division of Gastroenterology, Department of Internal Medicine, University Hospital of Patras, 26504 Patras, Greecechtriantos@hotmail.com (C.T.); 10 Department of Surgery, Transplantation and Gastroenterology, Semmelweis University, H-1082 Budapest, Hungary; 11 Department of Gastroenterology, Instituto de Investigación Sanitaria del Hospital de La Princesa, Hospital Universitario de La Princesa, Centro de Investigación Biomédica en Red de Enfermedades Hepáticas y Digestivas (CIBEREHD), Universidad Autónoma de Madrid (UAM), 28029 Madrid, Spain; 12 Division of Gastroenterology and Hepatology, Department of Internal Medicine, Medical University of Graz, 8036 Graz, Austria; 13 Hepato-Gastroenterology and Digestive Oncology, University Hospital CHU of Liège, B-4000 Liège, Belgium; 14 Department of Internal Medicine and Oncology, Semmelweis University, H-1083 Budapest, Hungarykislakpet99@gmail.com (P.L.L.); 15 Department of Gastroenterology, School of Medicine, Ankara University, 06100 Ankara, Turkey; 16 Department of Gastroenterology and Hepatology, University Hospital Centre Rebro, Day Hospital, Medical School, University of Zagreb, 10000 Zagreb, Croatia; 17 Department of Gastroenterology, Mater Dei Hospital, MSD 2090 Msida, Malta; 18 Department of Gastroenterology and Hepatology, Medical University of Silesia, 40-752 Katowice, Poland; 19 Department of Surgery, Albert Szent-Györgyi Medical School, University of Szeged, H-6725 Szeged, Hungary; 20 Department of Gastroenterology, Central Hospital of Northern Pest—Military Hospital, H-1062 Budapest, Hungary; 21 Division of Gastroenterology, McGill University Health Centre, Montreal General Hospital, Montreal, QC H3G 1A4, Canada

**Keywords:** Crohn’s disease, penetrating disease, abdominal abscess, percutaneous drainage, surgery

## Abstract

**Background**: Crohn’s disease complicated by intra-abdominal abscesses often requires surgery. Percutaneous drainage may prevent surgery, but optimal post-drainage management is unclear. We aimed to analyze the long-term outcomes of Crohn’s disease with intra-abdominal abscesses after intervention. **Methods**: Patients with penetrating Crohn’s disease and a single intra-abdominal abscess were enrolled in this multicenter, international, retrospective study after the detection of the abscess (baseline), with a minimum follow-up of 12 months. Those requiring urgent bowel resection were excluded. Patients were grouped by elective surgical need after successful (catheter insertion with effective drainage) percutaneous drainage (controls: no pre-resection drainage). The primary outcome was abscess recurrence. We also assessed stoma rate, post-procedural complications, hospitalizations, advanced treatment need, postoperative luminal recurrence, and need for re-drainage. **Results**: We studied 157 patients with Crohn’s disease (9 countries; males: 58%, median age: 32.4 [interquartile range: 25–39 years]); 89/157 underwent percutaneous drainage (median follow-up: 95.9 weeks [interquartile range: 58–104]). Abscess recurrence did not differ by drainage (*p* = 0.221). Abscess size was associated with advanced-treatment initiation (Odds ratio: 0.978; 95% confidence interval: 0.960–0.997, *p* = 0.023) and postoperative luminal recurrence (Odds ratio: 1.044, 95% confidence interval: 1.012–1.078, *p* = 0.007). Time to resection was longer after drainage, and ROC analysis raised predictive value for re-drainage (16.6 weeks post-drainage; AUC = 0.82, 95% confidence interval: 0.73–0.92). Patients without drainage had more post-procedural complications. **Conclusions**: Abscess size should guide management. Delayed resection may increase re-drainage odds, whereas surgery alone may have higher complication rates. Percutaneous drainage can safely postpone resection.

## 1. Introduction

Crohn’s disease (CD) is a chronic, immune-mediated, idiopathic inflammatory condition of the gastrointestinal tract characterized by ulcerative lesions, strictures, and penetrating complications along with several extraintestinal manifestations (EIMs), causing a high disease burden on patients’ lives and healthcare services [1].

Approximately 10–15% of patients with CD develop fistulizing complications during the disease course [2]. Patients with a more complicated disease course can have abdominal fistulas and abscesses that are often associated with inflammatory conglomerates or surrounding fluid collections, resulting in an extremely difficult-to-treat disease phenotype. Quality of life (QoL) is frequently poor and is influenced not only by luminal complaints but also by the disability resulting from the penetrating symptoms [3].

Management of intra-abdominal abscesses requires a multidisciplinary approach. The European Crohn’s and Colitis Organization (ECCO) guidelines suggest systemic immunosuppression, antibiotics, percutaneous drainage (PD) of the fluid collection, and surgical resection, but predictive factors to support decision-making are lacking. Systemic immunosuppression can be dangerous due to the increased risk of infections, while antibiotics may be insufficient. Surgical interventions are frequently associated with the risk of permanent stoma and short-bowel syndrome, in addition to septic complications. Thus, percutaneous drainage of an intra-abdominal abscess with a well-defined wall is currently recommended as first-line treatment, followed by a low threshold for elective resection [4,5].

Successful PD may prevent the need for emergency surgical resection in 14–35% of patients with an intra-abdominal abscess, thereby reducing the risk of acute surgery-related complications [6]. Furthermore, surgical resections might be unnecessary after successful image-guided PD in 30% of patients in the long-term (12–45 months), but there is limited evidence to support choosing the optimal management of patients with CD with intra-abdominal abscess who have undergone PD [4]. The available data regarding post-intervention outcomes are contradictory. Cohort studies and meta-analyses suggest that larger abscesses should be treated with surgery, but the complication rate is high. Predictors associated with successful non-surgical treatment and the optimal timing of surgical intervention after PD are lacking and often contradictory: abscess size, a higher Body Mass Index (BMI), presence of strictures, and recurrent abscesses are reported risks for surgery, whereas biological treatment can delay surgery [4,6,7,8]. Nevertheless, guidance on the optimal management after PD remains limited.

The primary study aim was to evaluate the long-term outcomes of CD complicated by intra-abdominal abscess after interventions (PD or resection alone). We also sought to identify factors associated with favorable long-term outcomes after successful PD and to compare the safety profiles of PD and surgical management.

## 2. Materials and Methods

### 2.1. Study Design and Participants

This international, multicenter, retrospective cohort study was conducted in 14 tertiary inflammatory bowel disease (IBD) referral centers in nine countries worldwide. Adult patients (age ≥ 18 years) with CD who presented with a simple >20 mm intra-abdominal abscess (Montreal B3) [9] between 1 January 2015 and 1 January 2024 were retrospectively identified and consecutively included. Medical records were reviewed between 1 January 2024 and 31 August 2025. Patients with multiple abscesses, previous bowel resection, or requiring emergency surgery were excluded from the analysis. Baseline was defined as the day when a single abdominal abscess with/without a fistula was first detected using abdominal computed tomography (CT), magnetic resonance imaging (MRI) or ultrasonography (US). Follow-up lasted ≥12 months. The patients were assigned to the PD group in case of successful PD performed by US guidance, whereas the patients not receiving PD due to technical, anatomical (e.g., anatomical location relative to adjacent organs, extent, multiloculated abscesses), or logistical infeasibility (availability of interventional radiologist) were assigned to the control group. The study protocol was reviewed by the ECCO Clinical Research Committee during the 8th and 9th National Study Group Meetings in 2023–2024. The reporting of this study conforms to the STROBE statement [10]. All patients provided their written informed consent to routine clinical care.

### 2.2. Data Collection

Data collection was based on existing medical record sheets, and the Research Electronic Data Capture (REDCap [11]) platform was used for standardized input into a uniform, anonymized form. The coordinating center (University of Szeged) was responsible for data integrity and safety.

The baseline demographic data (age, sex, date of birth, age at diagnosis, smoking status) and clinical data including (clinical activity per the Crohn’s Disease Activity Index, [CDAI] [12]), disease localization by Montreal classification [9], previous and current medications, EIMs), and details of abdominal abscess were recorded at the baseline. Characteristics of management choice (PD/versus elective resection) were recorded. Furthermore, abscess recurrence, hospitalization, requirement for re-drainage or new advanced treatment, stoma formation, luminal recurrence (Rutgeerts score [13]), and post-procedural complications were also recorded during follow-up.

### 2.3. Outcome Measurements and Definitions

The primary outcome was abscess recurrence during follow-up and in the first 3 months after intervention, defined as the development of an enhancing (>20 mm) fluid collection at the same site as the initial lesion after documented resolution (using US/CT/MRI imaging modalities according to the standard of care). The secondary outcomes were the need for a permanent intestinal stoma (defined as a need for a new permanent stoma during follow-up related to resection due to abdominal abscess), postoperative luminal recurrence (defined as Rutgeerts > i1), need for hospitalization (due to surgery, post-procedural complication, or disease activity), or new advanced treatment initiation. The need for re-drainage (defined as repeated PD of a fluid collection after documented resolution) was also analyzed as a secondary outcome. The tertiary outcome was the rate of post-procedural (PD and surgery) complications (defined as the occurrence of any complications ≤30 days after intervention).

### 2.4. Statistical Considerations

Due to the patient population characteristics, a power analysis was not performed since it included all eligible patients. The descriptive statistics are presented as the mean and standard deviation or the median and interquartile range (IQR) of the continuous variables, and categorical variables are presented as numbers and percentages. Normality was tested by using visual assessments (histograms and quantile–quantile plots). After checking the assumptions, the groups described with categorical variables were compared by chi-square or Fisher’s exact test, and continuous variables were compared by independent-samples *t*-tests. Due to the relatively small sample size in both cohorts, logistic regression models were constructed and adjusted for baseline clinical and sociodemographic differences between the groups; however, the limited number of events and residual confounding remain. Potential predictors associated with the primary and secondary outcomes were analyzed by univariable and multivariable Cox and logistic regression models. Study group, age, sex, smoking habits, disease duration, localization, perianal disease, baseline advanced treatment, steroid, and antibiotic use, baseline CDAI, C-reactive protein (CRP), procalcitonin, connecting fistula, resection, and abscess size were analyzed as possible covariates. Specifically, the variables in the univariable analysis with *p* < 0.20 were fitted and included in the multivariable analysis. Final multivariable models were obtained by backward selection using likelihood ratios, and odds ratios (OR) are shown. Log-rank tests were performed to determine the survival characteristics of time-dependent outcomes over time, whereas Kaplan–Meier curves were plotted. Regarding post-procedural complications, incidence ratios and overall patient year (PY) were calculated. We performed a complete case analysis to reduce bias and to achieve the most accurate description of the cohort. Bonferroni correction was used to reduce multiple comparisons error. A two-sided value of *p* < 0.05 indicated a statistically significant difference, and 95% confidence intervals (95% CI) were reported. IBM SPSS software (Windows, Version 29.0, IBM Corp., Armonk, NY, USA) was used to perform all statistical analyses.

## 3. Results

### 3.1. Patient Characteristics

One hundred and fifty-seven CD patients (male/female ratio: 0.58) were included in the analysis, with a median follow-up of 95.9 (IQR 58–104) weeks. The median age at inclusion was 32.4 (IQR 25–39) years, and the median disease duration was 9 years (IQR 6–18). More than half of the patients had never smoked, and approximately one-third were active smokers at baseline.

Nearly two-thirds of the patients had ileocolonic (59.9%) disease, and 27.4% presented with perianal manifestations. Arthralgia (or joint manifestation) was the most common EIM, occurring in 15.9% of the cases. At baseline, the majority of the cohort exhibited clinical or biochemical activity, with a mean CDAI score of 303.9 ± 106.5 and a mean CRP level of 88.1 ± 56.4 mg/L. Moderate endoscopic activity was observed in both study groups, with no significant difference between them (7.5 ± 5.8 vs. 11.3 ± 8.5).

Systemic corticosteroids and immunomodulators were used to treat 24.8% and 26.2% of the patients, respectively, whereas nearly half of the cohort was receiving any advanced treatment. Abdominal CT scans were performed in approximately three-quarters of the participants to assess the presence of intra-abdominal abscesses, and abdominal ultrasound and MRI were performed less frequently.

Overall, 89/157 (56.7%, 95% CI 48.9–64.4%) patients underwent PD after a median of 6 (IQR 1–16) weeks after baseline, and the controls were 68/157 (43.3%, 95% CI 35.6–51.1%) patients who underwent elective surgery without previous PD. Safe and proper drainage was technically impossible in all control-group patients. In the PD group, 62/89 (69.7%, 95% CI 60.1–79.2%) patients underwent elective resection during the follow-up period. The clinical and demographic characteristics were generally comparable between the groups, except for age [34.6 (IQR 27–45) vs. 30.1 (IQR 25–38) years, *p* = 0.01]. Further details of the baseline clinical and demographic characteristics are summarized in Table 1.

### 3.2. Effectiveness of Interventions and Predicting Outcomes

**Figure 1:**
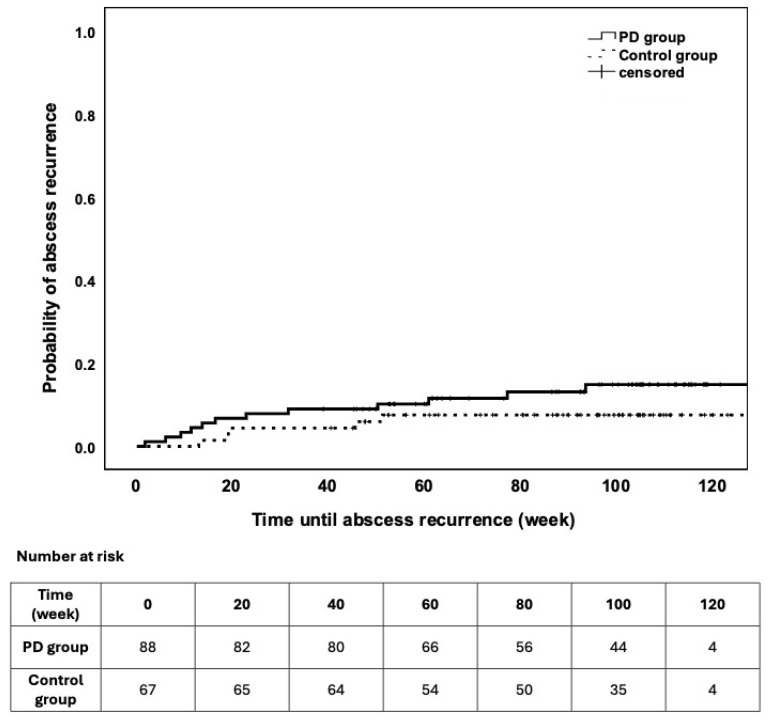
Survival characteristics of abscess recurrence during the follow-up. The patients were stratified into study groups. Differences were not observed regarding outcome (LogRank *p* = 0.221).

Abscess recurrence was observed in 12/89 (13.5%, 95% CI 6.4–20.6%) patients who had PD and in 5/68 (7.4%, 95% CI 1.1–13.6%) controls undergoing elective surgery without previous PD (χ^2^ = 1.500; *p* = 0.221) during the total follow-up period and in 4/89 (4.5%, 95% CI 0.2–8.8%) and 1/68 (1.5%, 95% CI 0–4.3%) after 3 months of intervention (*p* = 0.39), respectively. Furthermore, the time to recurrence did not differ between the groups (*p* = 0.21; Figure 1). No predictors of abscess recurrence were identified in our cohort.

**Figure 2:**
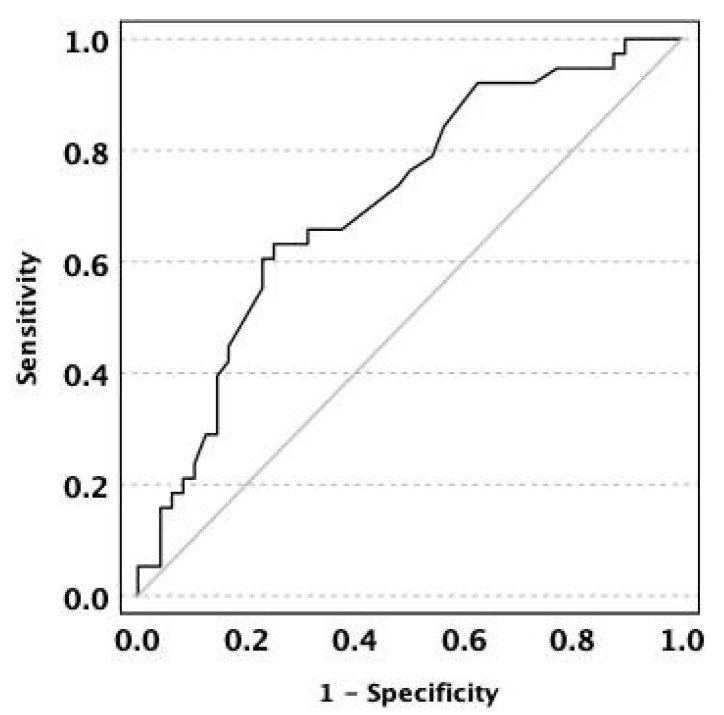
Receiver operating characteristic (ROC) curve demonstrating the discriminative capacity of abscess size for predicting postoperative luminal recurrence. The area under the curve (AUC) was 0.71 (95% confidence interval, 0.60–0.82). At the optimal cutoff value of 40.5 mm, the sensitivity and specificity were 66% and 63%, respectively.

Permanent ostomy was created in 5/62 (8.1%, 95% CI 1.3–14.8%) patients in the PD group and in 8/68 (11.8%, 95% CI 4.1–19.4%) patients in the control group (χ^2^ = 0.493; *p* = 0.48). No predictors of permanent stoma were identified. Postoperative luminal recurrence was more frequent in the control group [22/69 (31.9%, 95% CI 20.9–42.9%)] than in the PD group [16/89 (18.0%, 95% CI 10.0–26.0%); χ^2^ = 4.115; *p* = 0.043]. Recurrence was associated with the use of advanced therapy at baseline [OR 3.178; 95% confidence interval (CI) = 1.221–8.268; *p* = 0.018] and with larger abscess size (OR 1.044; 95% CI = 1.012–1.078; *p* = 0.007). Receiver operating characteristic (ROC) curve analysis demonstrated acceptable discriminative capacity of the abscess size for identifying postoperative luminal recurrence, with an AUC of 0.71 (95% CI 0.599–0.819). At the optimal cutoff (40.5 mm), the sensitivity was 66%, and the specificity was 63% (Figure 2).

**Figure 3:**
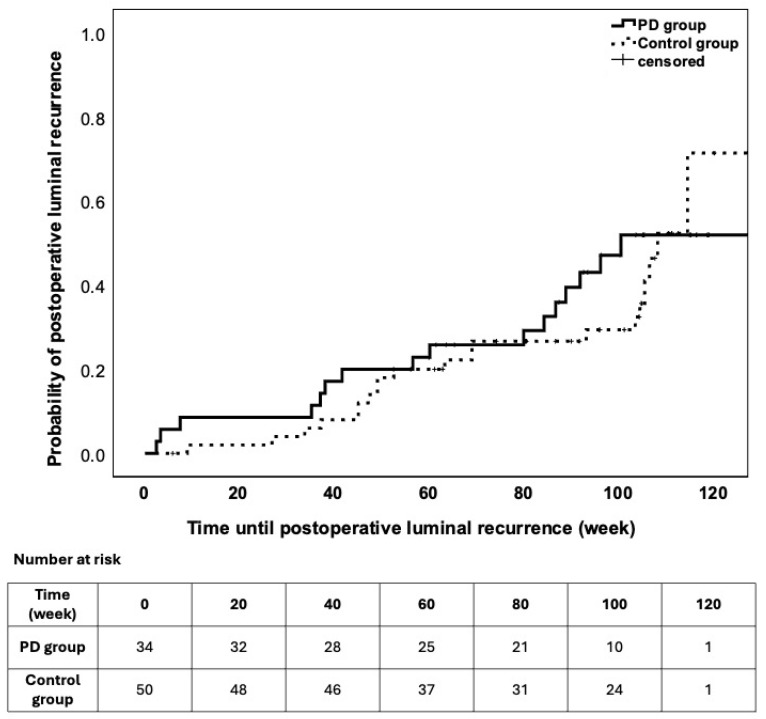
Survival characteristics of postoperative luminal recurrence during the follow-up. The patients were stratified into study groups. Differences in timing were not observed (LogRank *p* = 0.743).

The time until postoperative luminal recurrence did not differ between the groups (Figure 3).

**Figure 4:**
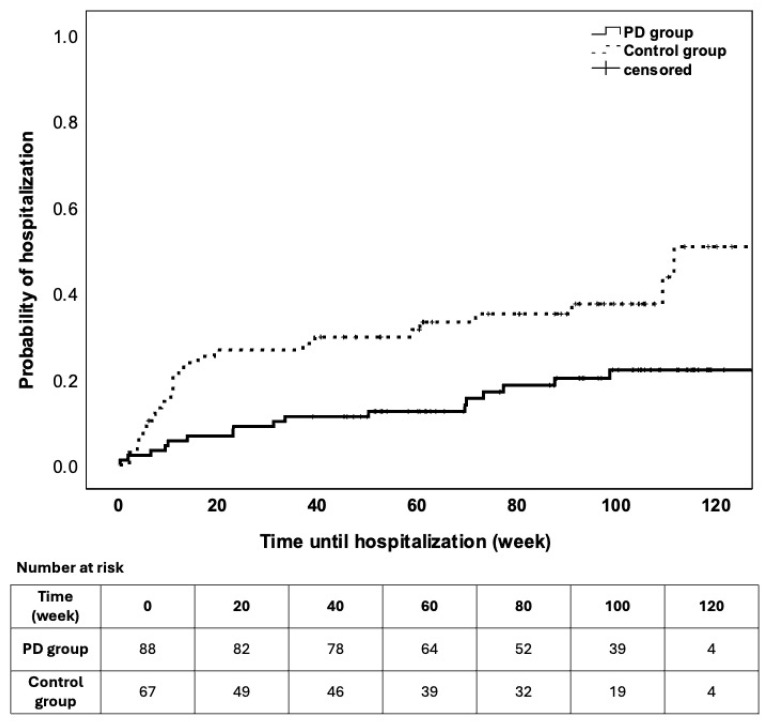
Survival characteristics of the need for hospital admission due to complications or disease activity during the follow-up. The patients were stratified into study groups. PD was coupled with less frequent hospital admissions (LogRank *p* = 0.004).

Hospital admission was more common in the controls [26/68 (38.2%, 95% CI 26.7–49.8%)] than in the patients with PD [17/89 (19.1%, 95% CI 10.9–27.3%), χ^2^ = 7.097; *p* = 0.008], and CRP was associated with an increased likelihood of hospitalization (HR of 1.006; 95% CI = 1.001–1.011; *p* = 0.025). Figure 4 shows the times until hospitalization for the groups.

New advanced treatment initiation was coupled with an increased baseline CDAI score (OR 0.002 95% CI, OR = 1.002–1.008; *p* = 0.002) and smaller abscess size (0.978; 95% CI OR = 0.960–0.997; *p* = 0.023), whereas the frequency did not differ between the groups [PD 46/89 (51.7%, 95% CI 41.3–62.1%) and control 34/68 (50.0%, 95% CI 38.1–61.9%), *p* = 0.834]. Re-drainage was observed in 14/89 (15.7%, 95% CI 8.2–23.3%) patients in the PD group, and no predictors were identified. Further details of the uni- and multivariable logistic regression analyses of different outcomes are presented in Table 2 and Supplementary Tables S1–S6.

### 3.3. Timing of Elective Resection

**Figure 5:**
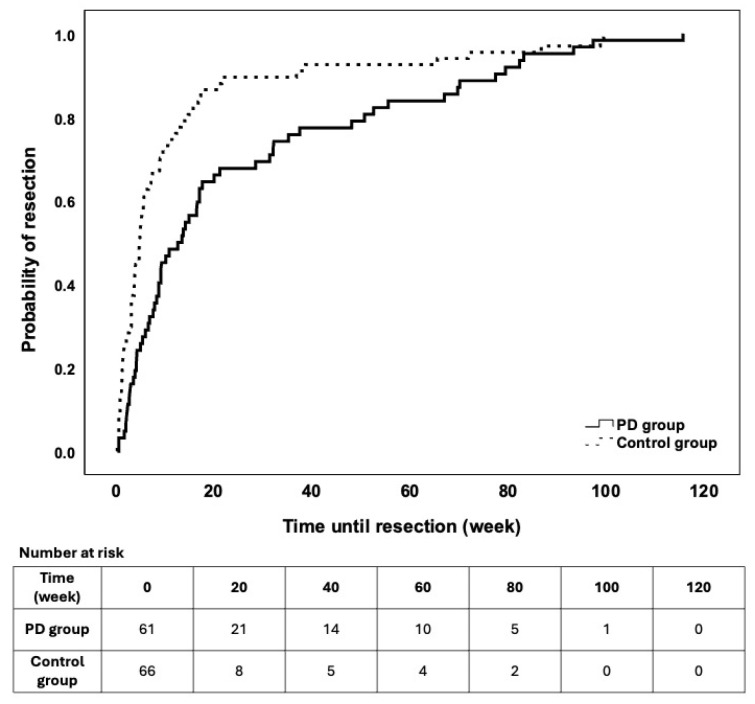
Survival characteristics of the need for elective resection during the follow-up. The mean number of weeks until elective resection was higher in the patients who received PD (25.4 ± 29.3) than in the Controls (12.4 ± 22.2, *p* = 0.003).

Overall, 62/89 (69.7%, 95% CI 60.1–79.2%) and 69/69 (100%, 95% CI 100.0–100.0%) patients underwent elective resection during the follow-up period in the PD and control groups after a mean of 25.4 (±29.3) and 12.4 (±22.2) weeks after detection of abdominal abscess (*p* = 0.003, Figure 5).

**Figure 6:**
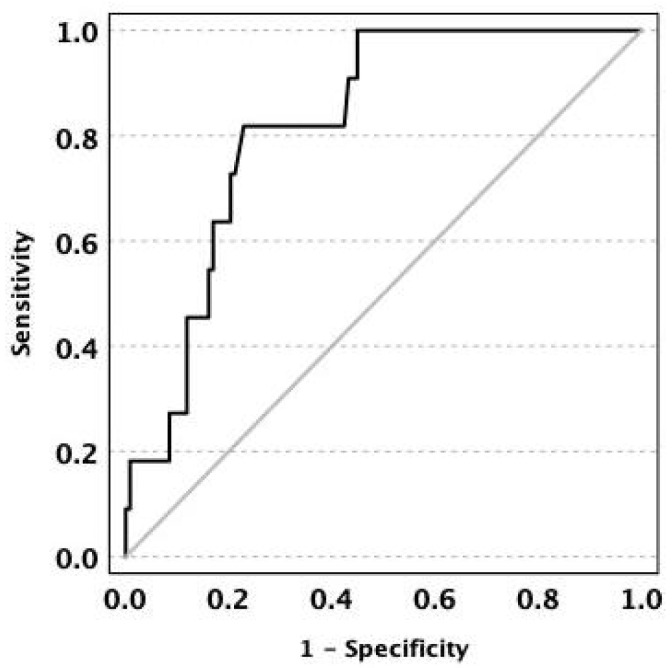
Receiver operating characteristic (ROC) curve demonstrating the discriminative capacity of time until resection for predicting the need for re-drainage. The area under the curve (AUC) was 0.82 (95% CI, 0.73–0.92). At the optimal cutoff value of 16.6 weeks, the sensitivity and specificity were 82% and 77%, respectively.

Among the variables examined, re-drainage in the PD group was associated with time until resection after PD (95% CI for OR = 1.010–1.046; *p* = 0.003). A ROC curve analysis demonstrated good discriminative capacity of time until resection for identifying the need for re-drainage, with an AUC of 0.82 (95% CI 0.73–0.92). At the optimal cutoff (16.6 weeks after PD), the sensitivity and specificity were 82% and 77%, respectively (Figure 6).

### 3.4. Analysis of Post-Procedural Complications

In total, 156.4 and 120.3 patient-years were recorded in the PD and control groups, respectively. Post-procedural complications were more frequent in the control group (8 of 68 patients [11.8%, 95% CI 4.1–19.4%], 6.7/100 PY) than in the PD group (3 of 89 patients [3.4%, 95% CI 0–7.1%], 1.9/100 PY; χ^2^ = 4.169, *p* = 0.04) during the follow-up. The most common complications were the development of new fistulas in the PD group and perforation or anastomotic leakage in the control group. Details of the post-procedural complications are summarized in Table 3.

## 4. Discussion

This international, multicenter retrospective study evaluated the long-term outcomes of CD complicated by intra-abdominal abscess, including 156 patients from 14 tertiary IBD centers worldwide. The abscess recurrence rates were comparable between the study groups; however, postoperative luminal recurrence (31.9% vs. 18.0%) and hospital admissions (38.2% vs. 19.1%) occurred more often in the control group, whereas the need for re-drainage was high among the patients who had PD (15.7%). Abscess diameter was associated with higher odds of postoperative luminal recurrence and initiation of new advanced therapy. Overall, 69.7% of the patients who had PD and all control patients underwent elective resection during a median follow-up of 95.9 weeks. Post-procedural complications were also more common in the control group.

In our cohort, abscess recurrence rates were comparable, in contrast to the findings of the meta-analysis by Clancy et al., which demonstrated a significantly increased risk of recurrence after PD (OR = 6.544) [4]. In the study by Deza et al., the risk of abscess recurrence was decreased after intestinal resection, especially after complete response, and was associated with an abscess located near the psoas muscle. The impact of strictures or fistulas was not seen [7]. Furthermore, abscess recurrence was higher after PD than after resection in the cohort described by Xie et al. [14], which is consistent with the study by Garcia et al. [15] as well. On the other hand, no statistically significant difference was seen between the initial PD and surgery groups in the study by Nguyen et al. [16]. These contradictory findings highlight the importance of patient characteristics and may indicate a more severe disease course in the PD group of our study, as reflected by the larger abscess sizes (mean: 51 mm) than those in the controls. In our analysis, no predictors of abscess recurrence were identified, including the use of biologicals, corticosteroids, or antibiotics, as well as clinical or biochemical activity markers and smoking status.

In a study by Deza et al. [7], a small proportion of patients with small (<30 mm) abscesses were able to manage with antibiotics alone, with outcomes similar to those for PD/surgical drainage, which highlights the importance of abscess size. Our study focused on the interventional treatment of abscesses; therefore, patients with antibiotic therapy were not enrolled. Furthermore, our cohort included a substantial number of patients with larger abscess sizes, which may help explain discrepancies between our outcomes and previously reported findings, reflecting a potentially more severe patient population.

Nevertheless, structuring disease, history of perianal disease, active ileal disease, and elevated BMI have been found to be associated with abscess recurrence in several studies, whereas biological treatment may decrease the risk [7,8,16]. Existing data are contradictory, as after adjustment for important confounders in the study by Lobatón et al., including abscess size, multilocularity, presence of fistula, and corticosteroid use, only initial PD remained an independent variable associated with treatment failure (OR 88.26) [17].

In our analysis, we adjusted for several potential confounders and covariates. Initial PD was associated with a decreased need for hospitalization, whereas higher CDAI scores predicted the initiation of therapy. Larger abscess size was associated with postoperative luminal recurrence, whereas the higher need for initiation of a new biological therapy observed in patients with smaller abscess sizes may reflect real-world positioning and utilization patterns of biological treatments, since reimbursement protocols were not evaluated in our study. The use of advanced treatment at baseline was also associated with postoperative luminal recurrence.

The need for new advanced therapy was similar between the groups; however, it was associated with higher baseline CDAI scores, which may help clinicians in decision-making regarding the initiation of new biologicals after intervention. The relatively high proportion of patients receiving systemic corticosteroids may be associated with worse clinical outcomes. It should be highlighted that Sun et al. found that initiation of new biological therapy was associated with a reduced risk of surgery [8]. The baseline age difference was not clinically significant, whereas the higher frequency of upper gastrointestinal involvement may indicate a more severe disease phenotype in the control group.

Postoperative luminal recurrence was more frequent in patients without previous PD and was associated with abscess size and the use of biologicals at baseline. A multicenter cohort study in 128 patients found an association between abscess size and treatment failure for medical treatment alone (AUC = 0.69), but an association with surgical or PD treatment was not observed [18]. In the present study, clinical recurrence was independent of medical, PD, or surgical treatment.

Despite existing literature and guidelines, there is still no consensus on how to guide clinical decision-making and determine the optimal timing of elective resection after successful PD. Sun et al. found that stricture and higher BMI were independent predictors of early resection (<1 year), whereas the use of biologicals and successful abscess resolution were associated with a reduced likelihood of early surgery [8]. In our cohort, the requirement for re-drainage was associated with a delayed resection after PD. A threshold of 16.6 weeks was identified, yielding a sensitivity of 82% and a specificity of 77% for predicting worse outcomes, thereby generating only hypotheses for future prospective studies.

In our study, the most pronounced difference between the two groups was observed in the rate of post-procedural complications, which was lower in the patients who underwent PD (3.4% vs. 11.8%). These findings are consistent with the findings of Deza et al., who found a 9.9% rate of post-procedural complications after PD, with an entero-cutaneous fistula observed as the most common complication [7]. A relatively high occurrence of entero-cutaneous fistula after PD was observed in our cohort as well. In a study by Xie et al., post-procedural complications were a little higher (20% vs. 69%), but PD also proved to be the safer approach [14]. On the one hand, Celentano et al. reported higher rates of postoperative and anastomotic leakage in patients with CD who underwent ileocecal resection after previous PD [19]. On the other hand, a recent meta-analysis concluded that there have been no significant differences in the complication rates between initial PD or surgery alone [4]. Furthermore, Liu et al. reported comparable complication rates after PD and surgery, of 24.0% and 25.8%, respectively, but no complications were observed when they used their trocar puncture technique [18]. It is important to emphasize that over time, the role of traditional surgical drainage has declined, whereas advances in radiological techniques have clearly contributed to reducing complication rates. To note, explicit assessment of several important surgical aspects (e.g., resection length, anastomosis type, and surgical approach) was not feasible, which may have contributed to an increased risk of postprocedural complications.

In our study, PD was associated with delayed resection, whereas primary surgery had a higher odds for post-procedural complications. Although ECCO recommends PD, a high number of patients in our cohort underwent surgery without previous PD (consecutive enrollment), possibly due to technical limitations, anatomical features, unavailability of interventional radiologists, or local institutional guidelines. Our findings support current recommendations that PD should be considered as a first-line approach in all suitable cases, as the resulting delay in resection may provide a safer pathway by controlling sepsis and reducing luminal disease activity, thereby improving wound healing. In the future, artificial intelligence may play an important role in predicting treatment response and enabling personalized treatment strategies [20].

Our study had several limitations. First, the consecutive enrolment of patients and the relatively small sample size resulted in limited statistical power, while the technical feasibility of PD and patient allocation to different groups and complete case analysis may have introduced selection bias. Second, as this was a multicenter international cohort, center-specific factors may have influenced patient management and outcomes. Third, the retrospective design precluded the establishment of firm causal relationships, while the relatively short follow-up period limited the ability to capture all relevant long-term clinical outcomes. Fourth, as the study focused exclusively on the effectiveness and safety of interventional treatments, the cohort represents a more severe population, potentially excluding patients successfully managed with conservative (non-interventional) treatment alone. Due to the limited number of outcome events relative to the number of candidate covariates, the multivariable models may be prone to overfitting. Unfortunately, it was not possible to control all technical, pharmacological, microbiological, and nutritional details of the interventions, which may have introduced bias. However, although several confounders were accounted for in the analyses, the study design and data collection methods did not allow adjustment for all potential sources of bias; therefore, the comparison between the groups should be interpreted as descriptive rather than causal. Finally, the ROC-derived cutoff values were not internally validated and may therefore be subject to optimism bias.

Despite these limitations, our study has several notable strengths. First, it represents one of the largest cohorts of CD patients with intra-abdominal abscesses undergoing initial intervention. Second, the international, multicentric study design minimized selection bias. Third, the relatively long follow-up period provided a valuable insight into the long-term disease course of penetrating CD parallel intervention. Finally, the use of pragmatic clinically relevant outcomes allowed us to make further hypotheses based on our findings for consideration in everyday clinical practice.

## 5. Conclusions

In conclusion, although delaying elective resection after PD may increase the risk of re-drainage, avoiding PD altogether may result in higher post-procedural complication rates. Therefore, PD could be considered to be the preferred initial approach in CD complicated by a single (large) intra-abdominal abscess. The abscess size and clinical outcome after PD may be considered to guide the timing of elective resection after successful PD, since PD can postpone surgery by controlling sepsis and reducing luminal disease activity. Elective resection may be considered after successful PD to minimize the risk of re-drainage. The abscess phenotype in CD remains a challenging and difficult-to-treat condition.

## Figures and Tables

**Table 1:** Baseline demographic and clinical characteristics of enrolled patients.

Variables	Total Cohort (*n* = 157)	PD Group (*n* = 89)	Control Group (*n* = 68)	Sig.
**Follow-up duration, ** *weeks, median (IQR)*	95.9 (58–104)	103.9 (76–112)	91.4 (77–104)	0.91
**Sex, *male*** *(%, 95% CI)*	91 (58.0, 50.2–65.7)	52 (58.4, 48.2–68.7)	39 (57.4, 45.6–69.1)	0.89
**Age at inclusion, ** *years, median (IQR)*	32.4 (25–39)	34.6 (27–45)	30.1 (25–38)	0.01
**Smoking habits at inclusion, ** *n (%, 95% CI)*				
never smoking	88 (59.9, 51.9–67.8)	51 (58.6, 48.3–69.0)	37 (61.7, 49.4–74.0)	0.48
active smoker	41 (27.9, 20.6–35.1)	23 (26.4, 17.2–35.7)	18 (30.0, 18.4–41.6)	
former smoker	18 (12.2, 6.9–17.5)	13 (14.9, 7.5–22.4)	5 (8.3, 1.3–15.3)	
*missing data*	10	2	8	
**CD disease duration at inclusion, ** *years, median (IQR)*	9 (6–18)	11.0 (6–17)	11.0 (7–18)	0.99
**Disease localization, ** *n (%, 95% CI) ^+^*				
ileal	55 (35.0, 27.6–42.5)	28 (31.5, 21.8–41.1)	27 (39.7, 28.1–51.3)	0.374
colonic	8 (5.1, 1.7–8.5)	6 (6.7, 1.5–12.0)	2 (2.9, 0–7.0)	
ileocolonic	94 (59.9, 52.2–67.5)	55 (61.8, 51.7–71.9)	39 (57.4, 45.6–69.1)	
upper GI involvement	19 (12.1, 7.0–17.2)	4 (4.5, 0.2–8.8)	15 (22.1, 12.2–31.9)	<0.001
**Perianal disease, ** *n (%, 95% CI)*	43 (27.4, 20.4–34.4)	29 (32.6, 22.8–42.3)	14 (20.6, 11.0–30.2)	0.10
**Extraintestinal manifestations, ** *n (%, 95% CI)*				
arthropathy	25 (15.9, 10.2–21.6)	13 (14.6, 7.3–21.9)	12 (17.6, 8.6–26.7)	0.18
skin disease	6 (3.8, 0.8–6.8)	4 (4.5, 0.2–8.8)	2 (2.9, 0–7.0)	0.67
eye disease	4 (2.5, 0.1–5.0)	3 (3.4, 0–7.1)	1 (1.5, 0–4.3)	0.48
hepatic disease	0 (0)	0 (0)	0 (0)	-
**Disease activity**				
CDAI, *mean (±SD)*	303.9 (106.5)	299.8 (119.5)	310.4 (114.5)	0.36
CRP, *mg/L, mean (±SD)*	88.1 (56.4)	109.9 (88.1)	106.6 (79.0)	0.81
SES-CD, *mean (±SD)*	9.9 (7.7)	7.5 (5.8)	11.3 (8.5)	0.11
**Conventional treatment at baseline, ** *n (%, 95% CI)*				
budesonide	10 (6.4, 2.5–10.2)	8 (9.0, 3.0–14.9)	2 (2.9, 0–7.0)	0.12
azathioprine	34 (21.7, 15.2–28.1)	16 (18.0, 10.0–26.0)	18 (26.5, 16.0–37.0)	0.20
methotrexate	7 (4.5, 1.2–7.7)	2 (2.2, 0–5.3)	5 (7.4, 1.1–13.6)	0.125
systemic corticosteroid	39 (24.8, 18.1–31.6)	18 (20.2, 11.9–28.6)	21 (30.9, 19.9–41.9)	0.13
**Advanced treatment at baseline, ** *n (%, 95% CI)*				
adalimumab	26 (16.6, 10.7–22.4)	12 (13.5, 6.4–20.6)	14 (20.6, 11.0–30.2)	0.23
infliximab	26 (16.6, 10.7–22.4)	14 (15.7, 8.2–23.3)	12 (17.6, 8.6–26.7)	
vedolizumab	11 (7.0, 3.0–11.0)	4 (4.5, 0.2–8.8)	7 (10.3, 3.1–17.5)	
ustekinumab	7 (4.5, 1.2–7.7)	1 (1.1, 0–3.3)	6 (8.8, 2.1–15.6)	
upadacitinib	5 (3.2, 0.4–5.9)	0 (0)	5 (7.4, 1.1–13.6)	
**Abscess diameter,** mm, *mean (±SD)*	51.8 (16.5)	51.3 (19.8)	43.3 (18.1)	0.06
**Imaging type, ** *n (%, 95% CI)*				
abdominal US	17 (10.8, 6.0–15.7)	7 (7.9, 2.3–13.5)	10 (14.7, 6.3–23.1)	0.21
CT	117 (74.5, 67.7–81.3)	71 (79.8, 71.4–88.1)	46 (67.6, 56.5–78.8)	
MRI	23 (14.6, 9.1–20.2)	11 (12.4, 5.5–19.2)	12 (17.6, 8.6–26.7)	

^+^ Montreal classification. Abbreviations: CD: Crohn’s disease, n: number of patients, IQR: inter-quartile range, SD: standard deviation of mean, CDAI: Crohn’s Disease Activity Index, PD: percutaneous drainage, CRP: C-reactive protein, MRI: magnetic resonance imaging, CT: computed tomography, US: ultrasonography, FU: follow-up.

**Table 2:** Multivariable logistic regression models to predict treatment effectiveness.

Outcomes	Variables	Sig.	O.R.	95% CI	
**Abscess recurrence**	*No significant predictor identified*				
**Need for ostomy**	*No significant predictor identified*				
**Postoperative luminal recurrence**	*Advanced treatment at baseline*	0.018	3.178	1.221	8.268
	*Abscess diameter*	0.007	1.044	1.012	1.078
**Need for hospitalization**	*Study group (PD)*	0.009	0.381	0.186	0.784
**Need for new advanced treatment initiation**	*CDAI*	0.002	1.005	1.002	1.008
	*Abscess diameter*	0.023	0.978	0.960	0.997
	*Fistula present*	0.073	0.531	0.265	1.062
	*Advanced treatment at baseline*	0.058	0.500	0.244	1.023

Abbreviations: Sig: significance level, O.R.: odds ratio (exp(B)), 95% CI: 95% confidence interval, FU: follow-up.

**Table 3:** Number and details of post-procedural complications.

	PD Group (*n* = 89)		Control Group (*n* = 68)	
	Event	IR per 100 PY (Total PY = 156.4)	Event	IR per 100 PY (Total PY = 120.3)
**Total number of complications**	3	1.9	8	6.7
**sepsis**	0	0	1	0.8
**hemorrhage**	0	0	1	0.8
**new fistula**	2	1.3	1	0.8
**perforation**	0	0	3	2.5
**catheter infection**	1	0.6	0	0
**anastomotic leakage**	0	0	2	1.7
**death**	0	0	0	0

Abbreviations: PY: patient years, IR: incidence rate, *n*: number of patients.

## Data Availability

TM is the guarantor of the article. All authors have read and agreed to the submitted version of the manuscript. The study data is available upon request to the corresponding author. To protect the privacy of the individuals who participated in the study, the data cannot be shared publicly. This manuscript, including the related data and figures, has not been previously published and is not under consideration elsewhere.

## Supplementary Materials

The following supporting information can be downloaded at: https://www.mdpi.com/article/10.3390/jcm15072724/s1, Table S1: Details of Cox proportional hazard models regarding abscess recurrence during follow-up. Table S2: Details of logistic regression models regarding permanent stoma need during follow-up. Table S3: Details of logistic regression models regarding postoperative luminal recurrence during follow-up. Table S4: Details of Cox proportional hazard models regarding need for hospital admission during follow-up. Table S5: Details of logistic regression models regarding need for new biological treatment initiation during follow-up. Table S6: Details of logistic regression models regarding re-drainage need during follow-up in PD group.

## Abbreviations

The following abbreviations are used in this manuscript:

AUC	Area under the curve
BMI	Body Mass Index
CD	Crohn’s disease
CDAI	Crohn’s Disease Activity Index
CRP	C-reactive protein
CT	Computed tomography
ECCO	European Crohn’s and Colitis Organization
IBD	Inflammatory bowel disease
MRI	Magnetic resonance imaging
OR	Odds ratio
PD	Percutaneous drainage
QoL	Quality of life
ROC	Receiver operating characteristic
