# Supplementary material for: Evaluation of Long-Term Outcomes of Crohn’s Disease Complicated by Intra-Abdominal Abscess: A Retrospective International Cohort Study
Source: J Clin Med. 2026 Apr 3;15(7):2724. doi: 10.3390/jcm15072724 (PMC13073424; doi:10.3390/jcm15072724)
Supplement: Supplementary file 1 [file jcm-15-02724-s001.zip › jcm-4186764-supplementary.pdf]

Supplementary data

Table S1. Details of Cox proportional hazard models regarding abscess recurrence during follow-up.

	Univariable				Multivariable			
	Sig.	H.R.	95% CI		Sig.	H.R.	95% CI	
**Study group (control)**	0.219	0.520	0.183	1.475				
**Age**	0.255	0.974	0.930	1.019				
**Sex (male)**	0.345	1.582	0.610	4.101				
**Smoking**	0.475	0.680	0.236	1.958				
**Disease duration**	0.706	0.988	0.927	1.053				
**Localization**[+]	0.671	0.898	0.547	1.475				
**Perianal disease**	0.472	1.440	0.532	3.895				
**Advanced treatment at baseline**	0.579	1.314	0.500	3.453				
**Systemic steroid use at baseline**	0.299	1.694	0.626	4.583				
**Antibiotic use baseline**	0.977	0.979	0.224	4.281				
**CDAI**	0.178	0.997	0.994	1.001	-	-	-	-
**CRP**	0.667	1.001	0.996	1.007				
**Procalcitonin**	0.831	0.955	0.626	1.457				
**Fistula present**	0.195	1.993	0.702	5.659	-	-	-	-
**Elective resection**	0.181	0.490	0.173	1.392	0.160	0.473	0.166	1.343
**Abscess diameter**	0.359	1.010	0.989	1.032				

Events per variable in final multivariable model: 17/3

[+]Montreal classification

Abbreviations: Sig: significance level, H.R.: hazard ratio, 95% CI: 95% confidence interval, CDAI: Crohn's Disease Activity Index, CRP: C-reactive protein

Table S2. Details of logistic regression models regarding permanent stoma need during follow-up.

	Univariable				Multivariable			
	**Sig.**	**O.R.**	**95% CI**		**Sig.**	**O.R.**	**95% CI**	
**Study group (control)**	0.157	1.835	0.792	4.254	0.157	1.835	0.792	4.254
**Age**	0.450	0.985	0.948	1.024				
**Sex (male)**	0.814	0.905	0.394	2.079				
**Smoking**	0.832	1.100	0.456	2.651				
**Disease duration**	0.596	1.014	0.962	1.070				
**Localization**+	0.450	1.191	0.757	1.872				
**Perianal disease**	0.668	0.812	0.312	2.108				
**Advanced treatment at baseline**	0.733	0.857	0.354	2.078				
**Systemic steroid use at baseline**	0.781	0.878	0.351	2.197				
**Antibiotic use baseline**	0.390	0.571	0.159	2.049				
**CDAI**	0.535	0.999	0.995	1.002				
**CRP**	0.696	1.001	0.996	1.006				
**Procalcitonin**	0.614	0.823	0.386	1.754				
**Fistula present**	0.599	1.250	0.545	2.869				
**Elective resection**	-	-	-	-				
**Abscess diameter**	0.720	1.004	0.983	1.026				

Events per variable in final multivariable model: 13/1

+Montreal classification

Abbreviations: Sig: significance level, O.R.: odds ratio (exp(B)), 95% CI: 95% confidence interval, CDAI: Crohn's Disease Activity Index, CRP: C-reactive protein

Table S3. Details of logistic regression models regarding postoperative luminal recurrence during follow-up.

	**Univariable**				**Multivariable**			
	**Sig.**	**O.R.**	**95% CI**		**Sig.**	**O.R.**	**95% CI**	
**Study group (control)**	0.813	0.901	0.379	2.141				
**Age**	0.925	1.002	0.964	1.041				
**Sex (male)**	0.481	0.728	0.301	1.759				
**Smoking**	0.796	0.890	0.369	2.148				
**Disease duration**	0.719	1.011	0.952	1.074				
**Localization**+	0.602	1.133	0.708	1.816				
**Perianal disease**	0.225	1.756	0.707	4.363				
**Advanced treatment at baseline**	**0.009**	3.326	1.349	8.199	**0.018**	3.178	1.221	8.268
**Systemic steroid use at baseline**	0.649	1.243	0.488	3.163				
**Antibiotic use baseline**	0.987	0.988	0.246	3.967				
**CDAI**	0.582	1.001	0.997	1.005				
**CRP**	0.810	0.999	0.994	1.005				
**Procalcitonin**	0.282	0.052	0.000	11.477				
**Fistula present**	0.112	2.077	0.843	5.119	-	-	-	-
**Elective resection**	-	-	-	-				
**Abscess diameter**	**0.004**	1.046	1.014	1.078	**0.007**	1.044	1.012	1.078

Events per variable in final multivariable model: 38/2

+Montreal classification

Abbreviations: Sig: significance level, O.R.: odds ratio (exp(B)), 95% CI: 95% confidence interval, CDAI: Crohn's Disease Activity Index, CRP: C-reactive protein, , -: the variable was dropped from the multivariable model

Table S4. Details of Cox proportional hazard models regarding need for hospital admission during follow-up.

	Univariable				Multivariable			
	Sig.	H.R.	95% CI		Sig.	H.R.	95% CI	
**Study group (PD)**	**0.005**	2.405	1.303	4.439	**-**	-	-	-
**Age**	0.985	1.000	0.973	1.027				
**Sex (male)**	0.930	0.973	0.531	1.784				
**Smoking**	0.675	1.147	0.604	2.177				
**Disease duration**	0.237	0.976	0.937	1.016				
**Localization**[+]	0.054	0.738	0.542	1.005	-	-	-	-
**Perianal disease**	0.591	0.829	0.418	1.644				
**Advanced treatment at baseline**	0.498	1.236	0.670	2.280				
**Systemic steroid use at baseline**	0.984	1.007	0.507	1.998				
**Antibiotic use baseline**	0.421	0.701	0.294	1.667				
**CDAI**	0.935	1.000	0.998	1.003				
**CRP**	0.111	1.003	0.999	1.007	**0.025**	1.006	1.001	1.011
**Procalcitonin**	**0.016**	1.203	1.035	1.398	-	-	-	-
**Fistula present**	0.417	0.780	0.429	1.420				
**Elective resection**	0.479	1.365	0.576	3.237				
**Abscess diameter**	0.525	0.995	0.978	1.011				

Events per variable in final multivariable model: 43/4

[+]Montreal classification

Abbreviations: Sig: significance level, H.R.: hazard ratio, 95% CI: 95% confidence interval, CDAI: Crohn's Disease Activity Index, CRP: C-reactive protein, -: the variable was dropped from the multivariable model

Table S5. Details of logistic regression models regarding need for new biological treatment initiation during follow-up.

	**Univariable**				**Multivariable**			
	**Sig.**	**O.R.**	**95% CI**		**Sig.**	**O.R.**	**95% CI**	
**Study group (control)**	0.834	0.935	0.497	1.758				
**Age**	0.243	0.984	0.957	1.011				
**Sex (male)**	0.905	1.039	0.551	1.959				
**Smoking**	0.437	0.769	0.397	1.491				
**Disease duration**	**0.079**	0.963	0.923	1.004	-	-	-	-
**Localization**[+]	0.170	1.264	0.905	1.766	-	-	-	-
**Perianal disease**	0.299	0.688	0.340	1.393				
**Advanced treatment at baseline**	**0.020**	0.450	0.230	0.881	0.058	0.500	0.244	1.023
**Systemic steroid use at baseline**	0.962	1.018	0.493	2.099				
**Antibiotic use baseline**	**0.044**	3.047	1.031	9.008	-	-	-	-
**CDAI**	**0.007**	1.004	1.001	1.007	**0.002**	1.005	1.002	1.008
**CRP**	0.437	0.998	0.995	1.002				
**Procalcitonin**	0.220	0.813	0.584	1.132				
**Fistula present**	0.063	0.547	0.289	1.033	0.073	0.531	0.265	1.062
**Elective resection**	**0.011**	3.158	1.296	7.694	-	-	-	-
**Abscess diameter**	**0.045**	0.982	0.966	1.000	**0.023**	0.978	0.960	0.997

Events per variable in final multivariable model: 80/6

[+]Montreal classification

Abbreviations: Sig: significance level, O.R.: odds ratio (exp(B)), 95% CI: 95% confidence interval, CDAI: Crohn's Disease Activity Index, CRP: C-reactive protein, -: the variable was dropped from the multivariable model

Table S6. Details of logistic regression models regarding re-drainage need during follow-up in PD group.

	Univariable				Multivariable			
	Sig.	O.R.	95% CI		Sig.	O.R.	95% CI	
**Age**	0.609	0.981	0.913	1.055				
**Sex (male)**	0.460	0.619	0.174	2.205				
**Smoking**	0.281	2.057	0.555	7.622				
**Disease duration**	0.188	0.929	0.833	1.037	-	-	-	-
**Localization**+	0.388	1.839	0.462	7.322				
**Perianal disease**	0.562	0.649	0.150	2.798				
**Advanced treatment at baseline**	0.045	3.794	1.029	13.981	-	-	-	-
**Systemic steroid use at baseline**	0.251	2.161	0.579	8.063				
**Antibiotic use baseline**	0.248	0.324	0.048	2.192				
**CDAI**	0.167	1.013	0.995	1.032	-	-	-	-
**CRP**	0.376	1.008	0.990	1.027				
**Procalcitonin**	0.216	.762	0.496	1.172				
**Fistula present**	0.897	1.156	0.128	10.413				
**Elective resection**	0.606	2.202	0.110	44.017				
**Abscess diameter**	0.076	0.907	0.814	1.010	-	-	-	-
**Time to PD (week)**	0.076	0.863	0.734	1.015	-	-	-	-

Events per variable in final multivariable model: 14/5

+Montreal classification

Abbreviations: Sig: significance level, O.R.: odds ratio (exp(B)), 95% CI: 95% confidence interval, PD: percutaneous drainage, CDAI: Crohn's Disease Activity Index, CRP: C-reactive protein
